# The Needs of Older People with Mental Health Problems: A Particular Focus on Dementia Patients and Their Carers

**DOI:** 10.1155/2012/638267

**Published:** 2012-10-24

**Authors:** Joaquim Passos, Carlos Sequeira, Lia Fernandes

**Affiliations:** ^1^Health Sciences, School of Health of Viana do Castelo, 4900-314 Viana do Castelo, Portugal; ^2^Nursing Unit, School of Nursing of Porto, 4200-072 Porto, Portugal; ^3^Clinical Neuroscience and Mental Health Department, Faculty of Medicine, University of Porto/Psychiatry of S. João Hospital Centre, 4200-319 Porto, Portugal

## Abstract

The problems and needs of older people are often associated with mental illness, characterized by a set of clinical manifestations, which constitute important domains for investigation and clinical practice. This paper presents the results of a pilot study whose main purpose was to identify met and unmet needs and to analyze the relationship between those needs, psychopathology and functionality in older people with mental health problems. A sample of 75 patients aged 65 or over, of both sexes, diagnosed with mental illness using ICD-9. The main diagnoses were depression (36%) and dementia (29.3%). Most patients had cognitive impairment (MMSE, 52%; CDT, 66.7%), depression (GDS, 61.3%), anxiety (ZAS, 81.3%), and moderate dependence (BI, 49.3% and LI, 77.3%). The main unmet needs found were daytime activities (40%), social benefits (13.3%), company (10.7%), psychological distress (9.3%), and continence (8%). The majority of these unmet needs occur with dementia patients. The majority of the carers of these patients had global needs (met and unmet) in terms of psychological distress. Findings also reveal that a low level of functionality is associated with dementia diagnoses. The association analyses suggest that dementia is an important determinant of the functional status and needs.

## 1. Introduction

The ageing of the population worldwide has been followed by an increase in the prevalence of mental illness, making this one of the most important causes of morbidity. In this context, there are numerous psychological and behavioural symptoms associated with dementia that strongly affect the objectives and life expectations of the elderly and increase the difficulties in the process of assessment, diagnosis, and treatment of these people [1].

Dementia occupies a central place as a condition of morbidity in this population, with a significant increase as we advance in age, constituting one of the most common causes of disability in western society [1–7].

The change in the age pyramid, which occurred mainly after the 50s, makes the study of ageing and old age a primary focus of attention and concern of health professionals and researchers, in order to deepen their knowledge and create evidence that will allow the development of more structured and targeted responses to meet specific needs of older people and to promote active and successful ageing.

As part of this work, elderly needs assessment is the fundamental base for a social/health policy definition and intervention. There is a met need if the person has a (moderate or serious) problem in the area for which they require assistance, which they receive and it is appropriate to met that need. There is an unmet need when individual has a significant problem in the area for which they are not receiving appropriate assistance (wrong kind of help or no assistance) [1, 8–11].

This paper aims to present the results of a pilot study conducted in the context of the Research Project ‘‘The needs of older people with mental health problems” developed under the Doctoral Program in Gerontology and Geriatrics in the Abel Salazar Biomedical Sciences Institute, University of Oporto/Aveiro, Portugal. The aim of the study was to identify the met and unmet needs of the elderly and their carers and to analyze the relationship between those needs, psychopathology and functionality in older people with mental health problems.

## 2. Materials and Methods

### 2.1. Participants

An elderly sample (over 65 years) of both sexes diagnosed with mental disorder according to the ICD-9—International Classification of Diseases [12] was recruited consecutively from the National Health Service in the Inpatient and Outpatient Department of Psychiatry and Mental Health (DSMP) at a Health Unit in Northern Portugal.

Also, 52 carers and 71 staff members were interviewed using the Camberwell Assessment of Need for the Elderly (CANE) to identify met and unmet needs.

It was established as inclusion criteria: ≥65 years old; mental disease (ICD-9 criteria) [12]; it was admitted as inpatients or outpatients in the Psychiatry and Mental Health Department (DSMP). The exclusion criteria were: blind/deaf and severe impairment in communication.

### 2.2. Instruments

The following instruments were used: the Camberwell Assessment of Need for the Elderly (CANE) [11]; the Minimental State Examination/MMSE [13]; the Clock Draw Test/CDT [14]; the Geriatric Depression Scale/GDS [15]; the Zung Anxiety Scale/ZAS [16]; the Barthel Index/BI [17]; the Lawton Index/LI [18]; and the Graffar's Social Classification/GSC [19].

In order to fit the assessment protocol to the context of the investigation, previously validated versions of the instruments or versions adapted to the Portuguese population were used. In that sense, the following were used: the Portuguese version of CANE [1]; the Portuguese version of the MMSE [20], with the new normative values adapted to the Portuguese population [21] (cognitive deficit: 0–2 school years ≤ 22; 3–6 school years ≤ 24; 7 or more school years ≤ 27). Regarding the Clock Draw Test, since a version adapted to the Portuguese population is still being developed, the original version was used [14] with the coding system proposed by Cacho et al. [22] (scores 0–10: >6 normal; ≤6 abnormal), which is being used by some institutions in Portugal. As to the Geriatric Depression Scale [23] (15 items version) and Zung Anxiety Scale [24], the Portuguese versions were used. Regarding the functionality, Portuguese versions of the Barthel Index [25] (scores 0–20: total independence 20; moderate dependence 13–19; severe dependence 9–12; total dependence 0–8) and Lawton Index [26] (scores 0–23: total independence 23; some level of dependence < 23) were also used. Finally, concerning the social classification, an adapted version of the Graffar's Social Classification [27] (scores 0–10: 0–2 class I; 3-4 class II; 5-6 class III; 7-8 class IV; 9-10 class V) was used.

### 2.3. Procedure

The participants were identified by the researcher through a contact with the mental health services. In the context of this study, the individuals were contacted and interviewed when they accessed the outpatient service or were inpatients during the study period. The researcher gave them information about the study and asked them if they wanted to participate. The researcher then tried to identify a suitable member of staff and a primary caregiver to perform the assessment.

After this, 21 inpatients, 46 outpatients, 6 in nursing homes, and 2 at home were assessed consecutively during the period from April to September 2011.

Despite the assessment being planned at the moment the patients had access to the health unit, in some of the cases the researcher had to go to their homes or nursing homes. Also, 52 carers and 71 staff members (psychiatrists, psychologists, social workers, and psychiatric nurses) were interviewed separately by the researcher using the Camberwell Assessment of Need for the Elderly (CANE). The CANE was given as a structured interview to the patients, their informal carer, staff, and evaluator from items 1 to 24, A and B sections. After the data collection, using the instruments and procedures listed above, the data was organized and classified and the statistical analysis was carried out using IBM SPSS, version 19.

In the development of this study, all procedures concerning ethical approval were obtained from the ethics committee and from the board of the institution where the study was held. All the necessary measures to safeguard participants' anonymity and confidentiality of information were also thoroughly followed.

## 3. Results and Discussion

The data was collected from a convenience sample of 75 patients, with ages ranging from 65 to 93 years (mean = 73.3 years and SD = 6.6). The majority were female (73.3%), married (49.3%), living with a partner (50.7%), and in rural areas (74.7%); a high percentage belonged to a very low social class (94.7%), according to the Graffar classification [19]. Most of them were interviewed as outpatients (73.3%), and the main diagnosis was depressive disorder (36%), followed by dementia (29.3%) and somatic comorbidity (98.7%). In 69.3% of the cases, there was an informal carer, and of these only 56% lived with the carer; most (80%) did not care for anyone else.

The main sociodemographic and clinical characteristics of the participants are presented in detail in Table 1.

Also, 52 carers with ages ranging from 21 to 80 years (mean = 58.9; SD = 15.02) were interviewed. The majority were male (51.9%), married (76.9%), a primary carer (94.2%), and patient's partner (50%).

The main sociodemographic characteristics of the carers are presented in Table 2.

### 3.1. Global Results of Applied Instruments

According to the global results of the applied instruments (Table 3), the majority of the patients had cognitive deficits in the evaluation with the MMSE (52%) and in the Clock Draw Test (66.7%). They also had high levels of depression (GDS, 61.3%) and anxiety (ZAS, 81.3%). As far as the degree of functionality is concerned, most had moderate dependence on basic activities of daily living (BADL) (BI, 49.3%) and some level of dependence on instrumental activities of daily living (IADL) (LI, 77.3%).

According to the identified needs of the patients, the results of the CANE on the evaluator's perspective (Table 4) show that the main met needs are related to physical health (93.3%), memory (81.3%), psychological distress (72%), information (70.7%), household skills (65.3%), eyesight/hearing (62.7%), and food (58.7%). The majority of unmet needs were found in the domain of daytime activities (40%), social benefits (13.3%), company (10.7%), psychological distress (9.3%), and continence sphincter (8%). None of the participants had unmet needs for caring for others, abuse/neglect and alcohol abuse. Also regarding the abuse/neglect, no participant registered any needs.

Concerning the carers' needs, on the evaluator's perspective, the results show that 45.3% had met needs and 6.7% unmet needs for psychological distress; another 21.3% had met needs for information.

### 3.2. Analysis of the Relationship between Psychopathology, Needs, and Functionality

Since the most common diagnoses were depression and dementia (which together represented 65.3% of the sample), some analyses were carried out to identify possible associations between these diagnoses and other sociodemographic and clinical variables. Those analyses were carried using chi-square (*x* ^2^) and point biserial correlation coefficient (*r*_pb_).

The results indicated a significant positive correlation between the medical diagnosis of dementia and depression and global needs (met and unmet) identified by CANE, from the perspective of carers, *r*_pb_ = .42, *P* < .05, staff, *r*_pb_ = .47, *P* < .01, and of the evaluator, *r*_pb_ = .46, *P* < .01. These findings reveal that a high level of global needs is associated with dementia diagnoses, while a slightly lower level of those same global needs is associated with depression.

The main unmet needs found, on the evaluator's perspective, were daytime activities, social benefits, company, and psychological distress. In the present study it was found that, in relation to daytime activities and benefits, the number of unmet needs is higher than those found in other studies [11, 28, 29]. In this view, it appears to be related to the fact that in Portugal there is an inadequate social network to address social problems and needs of older people with dementia or other mental health problems. The present Portuguese policies fall well short of the practice in other European countries, particularly in Northern Europe.

Moreover, in this sample, most patients lived in poor rural areas (74.7%), in a peripheral region of Northern Portugal, belonging to a very low socioeconomic class (94.7%), in contrast to the cited studies, where most patients were from urban or suburban areas [1, 8, 11]. In line with this, in the present study, it appears that the majority of the elderly (96%) had low education and lived alone or with a partner, of similar age and level of education. In most cases, this partner was designated as carer. Taking into account the low level of education, the poor rural areas where they live, and the family's typology (with only one or two elderly members), many of these people reported the lack of company as a significant problem. Also due to the lack of knowledge of rights and social benefits, the results of the CANE from the patients' perspective showed a lower level of unmet needs in daytime activities (12%) when compared to the evaluator' s perspective (40%). On the other hand, it remains also a policy difficulty concerning health and social services accessibility.

This highlights what has been reported in the literature regarding the management of care for patients with dementia, since the importance of systematically assessing needs and planning coordinated management of interdisciplinary care has been recognized. The importance is also assumed of ensuring a coverage that not only takes into account physical and environmental, but also psychological and social needs.

Regarding the psychological distress of the patients, the present results are in line with other studies in terms of global needs (met and unmet) [1, 8, 11, 28, 29], and the level of unmet needs is lower than those reported by these studies. This is probably related to the fact that in the present study a significant percentage of patients (26.7%) were psychiatric inpatients and 73.3% were psychiatric outpatients, in contrast to the majority of the referred studies [1, 8, 28, 29], where participants were evaluated mostly in the community or in an outpatient setting. This is also consistent with the Reynolds et al. study [11], where the level of unmet needs was lower than the previous studies.

Analyzing these results in relation to the dementia patients, it was found that the majority of unmet needs in daytime activities, social benefits, company, and psychological distress occurs in these participants, and the same is true for global needs (met and unmet). On the other hand, regarding functionality, the results show a significant negative correlation between the medical diagnosis of dementia and depression and the total scores obtained in Barthel, *r*_pb_ = −.37, *P* = .009, and Lawton Indexes, *r*_pb_ = −.49, *P* < .001, suggesting that low total scores in these two indexes are associated with dementia diagnosis, while slightly higher total scores are associated with depression diagnosis. These findings reveal that these patients present a wide range of needs in physical, psychological, and social areas that require great physical and psychological availability and good preparation by carers. This suggests that carers of patients with dementia are exposed to a greater demand, which can lead to higher levels of psychological distress.

With respect to the two items evaluated on the carers' needs, the majority had met or unmet needs in the domain of psychological distress, while in terms of information needs, this percentage is lower. Although there were no significant correlations between the diagnosis of dementia and the carers' psychological distress (*P* = .566), the high level of 59.1% of carers of the elderly with dementia, with global needs (met and unmet) in terms of psychological distress, is in line with the results obtained in most studies that suggest the existence of a high level of psychological distress in carers of patients with dementia, when compared to carers of patients with other chronic diseases [30–33]. In relation to the information and training needs of carers of elderly people with dementia, there is only a marginally significant association between the presence of these needs and the diagnosis of dementia, *X* ^2^(2) = 5.82, *P* = .055, verifying that, in this case, there is a tendency 41% of carers of elderly people with dementia registering global information needs. These data are also coherent with other studies that indicate that a high number of carers of people with dementia have information needs [34]. In this regard, other authors [35] state that in dementia lack of information may be a reason to use the health service, since many families surveyed do not understand executive deficits the meaning of an apraxia or delusional symptomatology.

## 4. Conclusions

This study shows a large deficit at the level of daytime activities associated with the progressive worsening of cognitive and functional deterioration in older people, resulting in the loss of levels of autonomy and the capability to satisfy their own needs. It also reveals a high prevalence of dementia in the elderly associated with a wide range of needs related to memory, personal safety, satisfying basic and instrumental daily living activities, and psychological distress (in both the elderly and the caregivers).

The high overall number of needs of the elderly with dementia and the high level of psychological distress of their carers highlight the importance of taking into account the suffering of carers and the need to provide supportive interventions to maintain their emotional well being and enable them to provide high quality care. Despite the attention paid to the role of families in the care of patients with dementia, carers still express low levels of knowledge about the disease and high levels of psychological distress. In this sense, much more must be done to improve information on the disease, training in appropriate skills for cognitive disorders and behavioural management, and possible psychological support.

The results also suggest the need to structure activities and interventions, maximizing the potential of each person, encouraging abilities, and preventing progressive deterioration of elderly people's skills in order to improve their independence and quality of life.

## Figures and Tables

**Table 1:** Sociodemographic and clinical characteristics of subjects.

Characteristics	*n* = 75
Age (years)	73.3 (SD = 6.6; range 65–93)
Gender *n* (%)	
Male	20 (26.7)
Female	55 (73.3)
Marital status *n* (%)	
Single	14 (18.7)
Married	37 (49.3)
Common law	1 (1.3)
Widowed	14 (18.7)
Divorced	8 (10.7)
Separated	1 (1.3)
Living situation *n* (%)	
Alone	12 (16.0)
With partner	38 (50.7)
With other relatives	15 (20.0)
With others	10 (13.3)
Status at interview *n* (%)	
Inpatient	20 (26.7)
Outpatient	55 (73.3)
Psychiatric diagnosis *n* (%)	
Depressive disorder	27 (36.0)
Dementia	22 (29.3)
Bipolar disorder	9 (12.0)
Schizophrenia and other psychoses	4 (5.3)
Alcohol dependence	4 (5.3)
Adjustment disorder	4 (5.3)
Anxiety	3 (4.0)
No diagnosis	2 (2.7)
Somatic comorbidity *n* (%)	
Yes	74 (98.7)
No	1 (1.3)
Socioeconomic classification (Graffar) *n* (%)	
Class IV—low	4 (5.3)
Class V—very low	71 (94.7)
Geographical area *n* (%)	
Urban	12 (16.0)
Suburban	7 (9.3)
Rural	56 (74.7)
Has a carer? *n* (%)	
Yes	52 (69.3)
No	23 (30.7)
Lives with carer? *n* (%) (*n* = 52)	
Yes	42 (56.0)
No	10 (13.3)
Is also a carer? *n* (%)	
Yes	15 (20.0)
No	60 (80.0)

**Table 2:** Sociodemographic characteristics of carers.

Characteristics	*n* = 52
Age (years)	58.9 (SD =15; range 21–80)
Gender *n* (%)	
Male	27 (51.9)
Female	25 (48.1)
Marital status *n* (%)	
Single	9 (17.3)
Married	40 (76.9)
Widowed	2 (3.8)
Divorced	1 (1.9)
Primary carer? *n* (%)	
Yes	49 (94.2)
No	3 (5.8)
Family carer *n* (%)	
Children	17 (32.7)
Husband/wife	26 (50.0)
Brother/sister	3 (5.8)
Other	2 (3.8)
Unrelated	4 (7.7)

**Table 3:** Overall results of the instruments applied.

Instruments	*n* = 75	Mean (SD)	Median	Range
MMSE: *n* (%)				
Total score		22.28 (6.45)	25.00	5–30
Without cognitive deficit	36 (48.0)			
With cognitive deficit	39 (52.0)			
Clock Draw Test: *n* (%)				
Total Score		4.65 (3.40)	4.00	0–10
Normal	23 (30.7)			
Abnormal	50 (66.7)			
No response	2 (2.7)			
GDS: *n* (%)				
Total score		8.14 (4.32)	9.00	0–15
No depression	28 (37.3)			
Depression	46 (61.3)			
No response	1 (1.3)			
Zung anxiety scale: *n* (%)				
Total score		48.49 (8.41)	49.00	25–69
No anxiety	13 (17.3)			
Anxiety	61 (81.3)			
No response	1 (1.3)			
Barthel index: *n* (%)				
Total score		17.52 (3.68)	19.00	3–20
Total independence	32 (42.7)			
Moderate dependence	37 (49.3)			
Severe dependence	3 (4.0)			
Total dependence	3 (4.0)			
Lawton index: *n* (%)				
Total score		13.41 (8.05)	15.00	1–23
Total independence	17 (22.7)			
Some level of dependence	58 (77.3)			

**Table 4:** Levels of need, as rated by the evaluator.

*n* = 75 (52 for A and B)	Needs identified *n* (%)			
Items/domains	No need	Met need	Unmet need	Not known
(1) Accommodation	60 (80.0)	12 (16.0)	3 (4.0)	—
(2) Household skills	21 (28.0)	49 (65.3)	5 (6.7)	—
(3) Food	26 (34.7)	44 (58.7)	5 (6.7)	—
(4) Self-care	39 (52.0)	31 (41.3)	5 (6.7)	—
(5) Caring for other	66 (88.0)	9 (12.0)	—	—
(6) Daytime activities	26 (34.7)	19 (25.3)	30 (40.0)	—
(7) Memory	12 (16.0)	61 (81.3)	2 (2.7)	—
(8) Eyesight/hearing	24 (32.0)	47 (62.7)	4 (5.3)	—
(9) Mobility	45 (60.0)	29 (38.7)	1 (1.3)	—
(10) Continence	45 (60.0)	24 (32.0)	6 (8.0)	—
(11) Physical health	2 (2.7)	70 (93.3)	3 (4.0)	—
(12) Drugs	51 (68.0)	21 (28.0)	3 (4.0)	—
(13) Psychotic symptoms	61 (81.3)	12 (16.0)	2 (2.7)	—
(14) Psychological distress	14 (18.7)	54 (72.0)	7 (9.3)	—
(15) Information	20 (26.7)	53 (70.7)	2 (2.7)	—
(16) Safety (deliberate self-harm)	57 (76.0)	16 (21.3)	2 (2.7)	—
(17) Safety (accidental self-harm)	59 (78.7)	11 (14.7)	5 (6.7)	—
(18) Abuse/neglect	75 (100)	—	—	—
(19) Behaviour	66 (88.0)	6 (8.0)	3 (4.0)	—
(20) Alcohol	71 (94.7)	4 (5.3)	—	—
(21) Company	34 (45.3)	33 (44.0)	8 (10.7)	—
(22) Intimate relationships	64 (85.3)	7 (9.3)	4 (5.3)	—
(23) Money	36 (48.0)	36 (48.0)	3 (4.0)	—
(24) Benefits	56 (74.7)	9 (12.0)	10 (13.3)	—
(A) Carer's need for information	35 (46.7)	16 (21.3)	1 (1.3)	23 (30.7)
(B) Carer's psychological distress	13 (17.3)	34 (45.3)	5 (6.7)	23 (30.7)
